# Progress in Surgery

**Published:** 1897-02-06

**Authors:** 


### PROGRESS IN SURGERY.

### SURGERY OF THE SPLEEN AND PANCREAS.

**The Treatment of Wandering Spleen** by splenorrhaphy is considered by Lykoff,[1] who thus summarises the results of his experiments: 1. By the employment of a catgut net it is possible to fix the spleen firmly and with certainty to the abdominal wall. 2. It is sufficient to fix the middle half of the organ. 3. The contraction of the newly-formed tissue contracts the spleen slightly. 4. The function is not altered. 5. The catgut threads play the principal rôle in the fixation, for, in place of them, connective tissue fibres form. 6. Any form of irritation or scarification of the surface of the spleen is harmful, as it does not materially increase the amount of fixation, and is apt to induce suppuration if the catgut has not been properly sterilised.

**Splenectomy**, according to the same author,[2] is indicated when the spleen is the seat of a primary and not secondary growth, which threatens to involve the entire system, and in cases where the pathological change is so great that the hope of a return of function cannot be entertained. In the case of a local lesion, splenotomy with partial resection should be employed in place of splenectomy. Steam should be employed to check hæmorrhage. Before performing splenectomy it is necessary to ascertain that the other hæmo-poietic viscera are in perfect functional activity.

**Rupture of the Spleen**, treated successfully by packing only for the stoppage of hæmorrhage, is reported by Pierson.[3] The patient was a boy, ten years of age, who had been thrown violently on his side. His worst pain was in the left axilla, and he vomited constantly. The following morning the abdomen was much distended and exquisitely tender. Pulse, 145; temperature, 103·5°; respiration, 40. Twenty hours after the injury a median incision was made, and large clots turned out of the abdominal cavity. After these had been washed away, the bleeding point was plainly seen in a transverse laceration of the hilum of the spleen. Owing to the depth of the cavity, and the nature of the tissue, it was found to be impossible to ligate the bleeding point. A long piece of iodoform gauze was therefore packed tightly in the rent, and brought out of the upper end of the abdominal wound. There was much shock following the operation. The gauze plugging was changed for the first time on the fifth day after the laparotomy. The patient's condition was considered too bad for splenectomy, which, no doubt, is a better surgical procedure in the majority of such cases. Erdman[4] mentions a similar treatment in the case of a gunshot wound of the spleen. He intended to perform splenectomy, but finding the patient's condition very bad, owing to five perforations of the small intestine and one of the omentum, he finally decided to simply pack the wound with gauze. The man, however, died six days after operation from acute uræmia.

**Pancreatic and Retro-peritoneal Cysts.**—Turner[5] records a typical case of pancreatic cyst occurring in a man thirty-four years of age. It was of rapid formation, and associated with considerable gastro-intestinal symptoms. The pain was apparently less than usual, and there was no history of injury. On admission to hospital there was a prominent, fluctuating, obviously cystic swelling in the epigastric, left hypochondriac, and umbilical regions. The abdomen was opened by an incision in the left linea semilunaris. The stomach and great omentum presented, but there was no appearance of cyst. Fluctuation could be felt behind the stomach, and an aspirator being passed through the omentum an inch below the greater curvature of the stomach, thirty-seven ounces of dark-coloured fluid were withdrawn. The opening was enlarged, and it was evident that the stomach and omentum were adherent to the front of the cyst wall. The opening into the cyst was stitched to the abdominal parietes, and a drainage tube inserted. The tube was removed a month after operation, the patient making a good recovery. The fluid was alkaline, and had a specific gravity of 101·4. Its dark brown colour was probably due to methæmaglobin. No bile pigment was found. There was a large amount (half) of albumin present; and the fluid contained albumose, but no peptone. In referring to this case, the author strongly condemns aspiration previous to abdominal incision, as it is fraught with much danger owing to the anatomical position of the cyst. A case of retro-peritoneal sanguineous cyst is reported by Penrose and Turner,[6] in a man fifty-six years of age, who had noticed an abdominal swelling about a year after being kicked in the abdomen by a donkey when ten years of age. It occupied the hypogastric region chiefly, and was operated upon in a somewhat similar manner to the case just recorded, but the patient died five weeks afterwards. Before operation the patient suffered from wasting, feebleness, constant pain, and nocturnal temperature, which would have led sooner or later to a fatal issue, and made operation the only means of relief. Post-mortem the cyst was found to grow from the pouch between the bladder and rectum, and appeared under the peritoneum over the upper border of the prostate. It was, however, quite distinct from the prostate. In connection with this case the author quotes Fisher's[7] paper, in which some twenty cases are collected. Only one of these is completely retro-peritoneal, seven being partially so. In only six is there a history of traumatism. A considerable interval of time between the injury and the formation of the cyst is constantly seen, and this induced Fisher to suggest that the cysts may be due to injury done to the large nervous plexuses rather than a direct lesion of the vessels. Aspiration or tapping has been successful in four out of eight cases. The patients have died in two cases out of four in which tapping has been followed by the injection of fluid, and similar results have followed extirpation. Laparotomy and stitching the cyst wall to the abdominal parietes has been successful in two cases.

[1] Internat. Med. Mag., May, 1896. [2] Loc. cit. [3] New York Med Jour., Aug. 22, 1896, p. 272, [4] Ibidem, p. 273. [5] Lancet, July 4, 1896, p. 26. [6] Ibidem, p. 28. [7] Guy's Hosp. Reports, Vol. XLIX.

